# Real-world effectiveness and safety of rituximab and reduced-dose CHP with polatuzumab vedotin (pola-R-CHP) in patients aged > 80 years with diffuse large B-cell lymphoma: a retrospective analysis

**DOI:** 10.1007/s44313-025-00059-5

**Published:** 2025-02-05

**Authors:** Shuku Sato, Shun Tsunoda, Wataru Kamata, Tomiteru Togano, Yotaro Tamai

**Affiliations:** Division of Hematology, Shonan Kamakura General Hospital, 1370-1 Okamoto, Kamakura, Kanagawa 247-8533 Japan

**Keywords:** Older patients, Diffuse large B-cell lymphoma, Polatuzumab vedotin, POLARIX trial, Pola-R-CHP, Reduced-dose chemotherapy, Octogenarian

## Abstract

**Purpose:**

The efficacy and safety of polatuzumab vedotin combined with rituximab, cyclophosphamide, doxorubicin, and prednisolone (pola-R-CHP) in patients aged ≥ 80 years with untreated diffuse large B-cell lymphoma (DLBCL) remain largely unexplored.

**Methods:**

In this study, we administered a reduced-dose pola-R-CHP regimen to 38 patients with DLBCL aged > 80 years. Extending the findings of the POLARIX trial in this older individuals’ cohort, we conducted a retrospective analysis to assess the efficacy and safety of the treatment in a real-world clinical setting.

**Results:**

After 12 months, the overall and progression-free survival rates were 86.2% (95% confidence interval [CI]: 70.0–94.0) and 78.5% (95% CI: 59.2–89.5), respectively. Although the incidence of febrile neutropenia was relatively high (32%), an increased risk was observed in patients with an average relative dose intensity of < 70%, even with reduced treatment intensity. Notably, none of the patients required a dose reduction of polatuzumab vedotin owing to peripheral neuropathy.

## Introduction

The standard treatment for patients with diffuse large B-cell lymphoma (DLBCL), the most common type of mature B-cell lymphoma [1], includes rituximab, cyclophosphamide, doxorubicin, vincristine, and prednisolone (R-CHOP) [2]. However, the POLARIX trial indicated that polatuzumab vedotin (Pola) combined with rituximab, cyclophosphamide, doxorubicin, and prednisolone (pola-R-CHP) resulted in a lower risk of disease progression and relapse than R-CHOP [3]. For the CHOP regimen, reducing the dose of chemotherapeutic agents and extending the treatment duration, particularly in older patients, those with organ dysfunction, and those experiencing infections, is often necessary. In addition, social factors such as limited access to care or support can lead to prolonged treatment intervals.

The R-mini-CHOP regimen, which includes a 50% reduced dose of anthracycline and cyclophosphamide, was effective for most older patients in several clinical trials, especially for patients aged > 80 years [4, 5]. Several studies on reduced-dose R-CHOP regimens have shown that maintaining a high relative dose intensity (RDI) enhances response rates and prolongs overall survival (OS) and progression-free survival (PFS) [6, 7].

Although older patients often receive a reduced dose of R-CHOP, the POLARIX clinical trial primarily included patients aged < 80 years, resulting in the efficacy and safety of Pola for untreated DLBCL in patients aged > 80 years being unproven. In addition, although a reduced-dose pola-R-CHP regimen may improve outcomes in older patients with DLBCL, its efficacy in this population remains unclear.

We extended the findings of the POLARIX study to patients aged ≥ 80 years by introducing reduced pola-R-CHP in a clinical setting and conducted a retrospective observational study to evaluate its efficacy and safety.

## Methods

We retrospectively analyzed 38 patients aged > 80 years with DLBCL who were treated with pola-R-CHP at the Shonan Kamakura General Hospital between September 2022 and February 2024. All patients received at least one cycle of chemotherapy, including those who discontinued treatment early owing to toxicity or disease progression. The RDI of all patients was monitored.

The pola-R-CHP regimen consisted of prednisolone (100 mg/day orally on days 1–5), doxorubicin (50 mg/m^2^ intravenously on day 1), cyclophosphamide (750 mg/m^2^ intravenously on day 1), Pola (1.8 mg/m^2^ intravenously on day 1), and rituximab (375 mg/m^2^ intravenously on day 1), administered every 21 d for six cycles. Dose adjustments were made at the discretion of the attending physicians. The RDI was calculated based on a planned treatment cycle of 21 d. Dose intensity (DI) was defined as the scheduled dose per cycle (mg/m^2^) divided by the planned duration per cycle (weeks). The RDI (%) was determined by dividing the actual dose intensity by the target dose intensity and multiplying the result by 100. The average RDI (ARDI) represented the RDI delivered for each chemotherapeutic agent (doxorubicin and cyclophosphamide) across all treatment cycles. It was calculated by dividing the total actual dose (mg/m^2^) over the total treatment duration (weeks) by the total planned dose (mg/m^2^) over the planned treatment duration. In cases with fewer than six cycles due to disease progression or death, the number of regimen cycles administered without any reduction or delay was regarded as the maximum ARDI value of 100%.

Patients with central nervous system (CNS) involvement at initial presentation and those receiving radiation therapy before chemotherapy were excluded. The requirement for informed consent was waived for this retrospective study. All patient information was confirmed before data analysis. In accordance with the simplified frailty score [8], a geriatric assessment was performed before administering pola-R-CHP therapy using three tools: ADL, Charlson Comorbidity Index (CCI), and Geriatric Nutritional Risk Index (GNRI). The CNS-International Prognostic Index (CNS-IPI) includes patients aged > 60 years, LDH > normal, performance status > 1, Ann Arbor Stage III or IV, extranodal involvement, and kidney and/or adrenal gland involvement assessed; each factor was scored one point if present or zero if not present, with a score of 4–6 points defined as high risk [9]. This study adhered to the Declaration of Helsinki, and the protocol was approved by the Ethics Committee of Shonan Kamakura General Hospital on June 4, 2024 (TGE2422-024).

Specific diagnostic criteria for DLBCL included pathologically confirmed diffuse infiltration of large, atypical lymphocytes that were CD20 positive, CD3 negative, and demonstrated Ki67 staining of ≥ 80%. Cases that were FISH-positive for MYC and BCL2 were classified as high-grade B-cell lymphomas. The primary endpoint of the study was OS, which was assessed unadjusted and adjusted for treatment and baseline prognostic factors. OS was measured from the date of inclusion to the date of death, irrespective of the cause.

### Statistical analysis

OS was calculated from the initiation of chemotherapy to the time of death or last follow-up. PFS was defined as the time from the initiation of chemotherapy to relapse, disease progression, death, or the last follow-up. The Kaplan–Meier method was used to estimate OS and PFS rates. The *p*-value was computed using the log-rank test, and statistical significance was set at *p* < 0.05. Cox regression analysis was used to calculate the odds ratios in both univariate and multivariate contexts. Cutoff values for dichotomizing risk factors were determined using receiver operating characteristic (ROC) curve analysis, selecting the optimal cutoff point based on the shortest orthogonal distance to the ideal value on the ROC curve. The optimal RDI cutoff value (0.7) was derived using the Cox proportional hazards model for OS, using time-dependent ROC analysis (for analysis) and the ggplot2 package (for visualization). An ARDI cut-off value of 70% was used. All analyses were conducted using EZR (Saitama Medical Centre, Jichi Medical University; http://www.jichi.ac.jp/saitama-sct/SaitamaHP.files.statemedEN.html; Kanda, 2012), a graphical user interface for R (The R Foundation for Statistical Computing, version 2.13.0), which includes a modified version of R Commander (version 1.6–3) designed to add statistical functions [10].

## Results

The patient characteristics are summarized in Table 1. The median age was 84.3 years (range: 80–95 years), and eight patients (21%) had an Eastern Cooperative Oncology Group performance status (PS) of ≥ 2 upon arrival. Most patients were diagnosed with DLBCL (37 patients, 97%), whereas one patient had high-grade B-cell lymphoma with MYC and BCL2 rearrangements. Stage III–IV disease was present in 30 patients (79%), with 16 patients (42%) having high-risk IPI scores and four patients (10%) having high-risk CNS-IPI scores.

**Table 1:** Baseline characteristics of the patients enrolled in the study

Characteristics	*n* = 38	%
Median age (range)	84.3 (80–95)	
80–84	23	(61)
85–90	12	(31)
≥ 90	3	(8)
Sex (number of participants)		
Male	20	(53)
Female	18	(47)
ECOG performance status (no.)		
0–1	30	(79)
≥ 2	8	(21)
Ann Arbor stage		
I-II	8	(21)
III-IV	30	(79)
Serum lactate dehydrogenase level (normal range: 124–222 U/L)	764 (188–9421)	
Elevated lactate dehydrogenase level ≥ 223U/L	27	(71)
Bulky disease	8	(21)
CCI ≥ 2	6	(16)
Albumin < 3.5 g/dL	27	(71)
Hb < 12 mg/dL	24	(63)
ADL < 5	12	(32)
IADL < 7	17	(45)
Body weight at starting chemotherapy (kg)	51.0 (36–76)	
IPI-score		
Low (1)	3	(8)
Intermediate (2–3)	19	(50)
High (4–5)	16	(42)
Geriatric nutritional risk index		
< 82	16	(42)
82 to < 92	11	(28)
92 to 98	4	(11)
> 98	7	(18)
Simplified frailty score		
Fit	7	(18)
Unfit	12	(32)
Frail	19	(50)
Pathology		
DLBCL	37	(97)
GCB	15	(39)
non GCB	22	(58)
High grade B cell lymphoma	1	(3)
CD5 positive	5	(13)
prophylactic G-CSF	22	(57)
Number of cycles-median (range)	5.3 (1–6)	
1 course	3	(8)
3, 4 courses	8	(21)
5 courses	3	(8)
6 courses	24	(63)
2 courses of rituximab maintenance therapy after Pola-R-CHP		
yes	12	(32)

*ECOG* Eastern Cooperative Oncology Group, *CCI* Charlson comorbidity index, *IPI* International Prognostic Index

The median number of treatment cycles was five (range: 1–6). Twenty-four patients (63%) completed all six courses, whereas 14 patients (37%) did not. Among the 14 patients who did not complete therapy, the reasons included one case of disease progression, three cases of bacterial infection, two cases of fatigue, one case of coronavirus (COVID)−19, one case of sudden death owing to unknown origin in a dialysis patient, two cases of dementia, and four patients who discontinued treatment based on the recommendation of the physician owing to localized stage and low-risk IPI. Three patients completed only one course, with discontinuation reasons being dementia and fatigue in two cases and bacterial sepsis with underlying liver cirrhosis in one case.

Responses were evaluated for all patients at the end of the first-line treatment. Among these patients, 32 (84%) achieved a complete response, four (10%) showed a partial response, one (2%) had stable disease, and one (2%) experienced progressive disease.

**Fig. 1:**
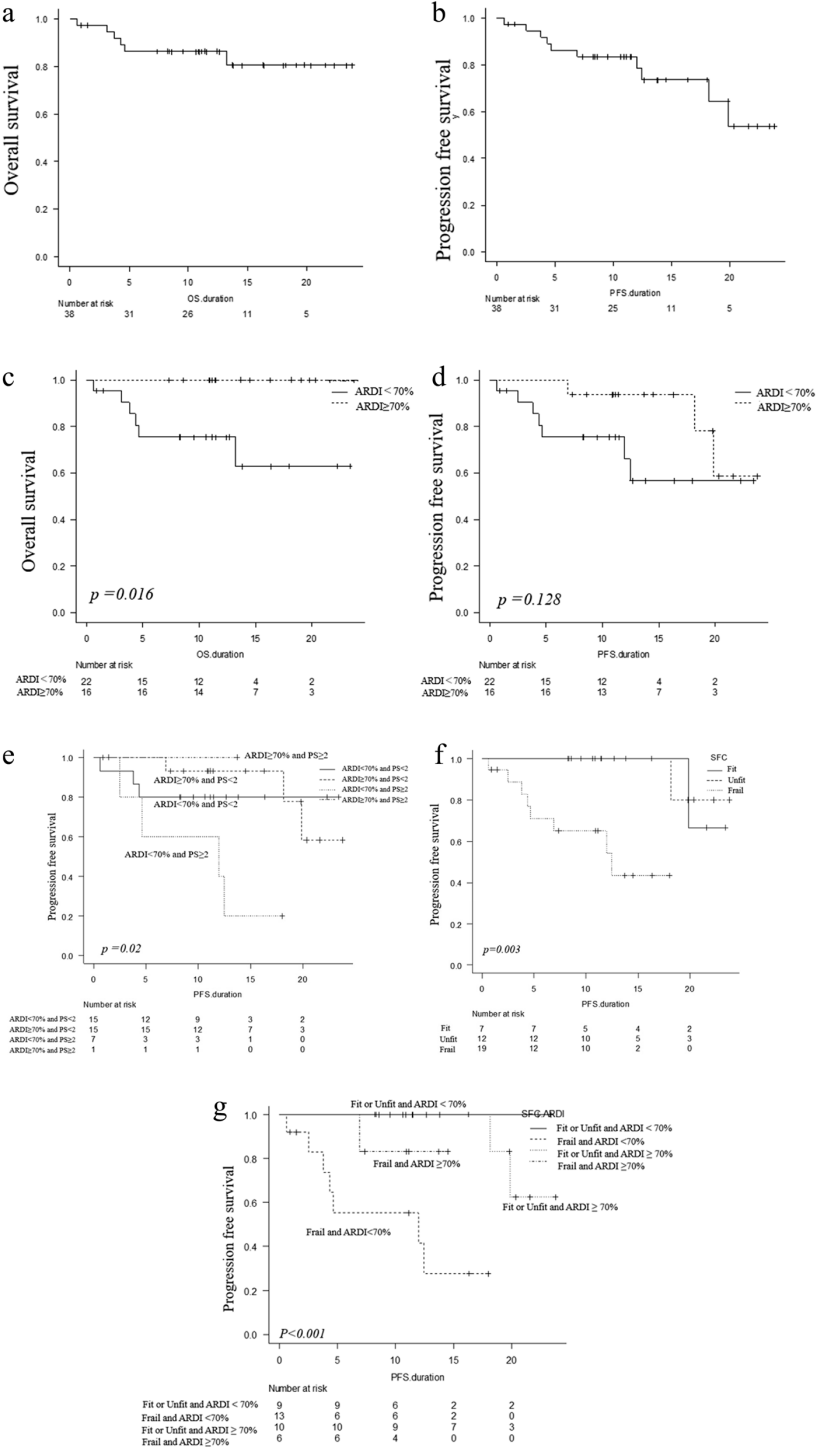
Overall survival (**a**) and progression-free survival (**b**) of 38 very elderly patients with diffuse large B cell lymphoma treated by Pola-R-CHP. Comparison of 12-month overall survival (**c**) and progression-free survival (**d**) between patients with average relative dose intensity (ARDI) ≥ 70% and < 70%. ARDI values were stratified by ECOG PS ≥ 2 or PS < 2, patients with PS ≥ 2 ARDI < 70% had significantly inferior PFS (**e**). According to the simplified frailty score, which assesses frailty, PFS at 12 months was significantly worse in frail (**f**). Frail and an ARDI < 70% had the worst PFS, but there was no difference in PFS between fit and unfit patients, regardless of the ARDI status (**g**)

The median follow-up duration was 11.6 months (range: 1–24). The OS and PFS rates at 12 months were 86.2% (95% CI: 70.0–94.0) and 78.5% (95% CI: 59.2–89.5), respectively (Figs. 1a and b).

We established an ARDI cutoff of 70% based on ROC curve analysis. Sixteen patients had ARDI ≥ 70% and twenty-two patients had ARDI < 70%. The OS rate at 12 months was significantly higher in patients who maintained an ARDI of ≥ 70% (ARDI ≥ 70% vs. ARDI < 70%; 100% [95% CI; NA-NA] vs. 75% (95% CI; 50.5–88.9), *p* = 0.015). Similarly, these patients had a higher PFS rate at 12 months (ARDI ≥ 70% vs. ARDI < 70%; 93.8% [95% CI: 63.2–99.1] vs. 65.9, [95% CI: 37.1–84.0], *p* = 0.12) (Figs. 1c and d). Patients with PS ≥ 2 and ARDI < 70% had significantly inferior PFS (Fig. 1e). We also analyzed PFS based on the simplified frailty score [8], dividing patients into three groups: fit (seven cases), unfit (12 cases), and frail (19 cases), based on age, ADL, CCI, GNRI, and age < 85 years. The PFS rate at 12 months was significantly better for the fit and unfit groups than for the frail group: fit, 100%; unfit, 100%; frail, 54.3% (95% CI: 25.1–75.3) (*p* = 0.003, Fig. 1f). Patients who were frail with ARDI < 70% had the worst PFS (*p* < 0.001); however, no difference in PFS between fit and unfit patients was observed irrespective of ARDI status (*p* = 0.37).

The CNS-IPI indicated high risk and PS 0–1 in two patients; intrathecal MTX was administered for each course of pola-R-CHP therapy, but in all cases, high-dose MTX for CNS relapse prophylaxis was not administered. CNS relapse was observed in two of the 38 patients, both with high risk based on CNS-IPI; however, prophylactic IT could not be performed owing to poor PS. We did not perform radiation on any patients who underwent positron emission tomography-complete response, including those with bulky disease. Seven patients with bulky disease achieved a complete response. One patient developed progressive disease during the course; therefore, no additional radiation therapy was performed [10].

To investigate prognostic factors, we conducted univariate and multivariate Cox regression analyses. Univariate analysis identified age ≥ 85 years, patients with frailty based on simplified frailty score, GNRI, and an ARDI < 70% as independent prognostic factors for OS. However, the multivariate analysis did not show statistically significant differences (Table 2).

**Table 2:** Univariate and multivariate Cox hazard regression analysis of OS

	Univariate analysis			Multivariate analysis		
	Odds ratio	95%CI	*p* value	Odds ratio	95%CI	*p* value
Age ≥ 85	9.97	1.14–87.3	0.04	1.99	0.19–17.4	0.58
bulky disease	1.99	0.36–10.96	0.42			
Performance status ≥ 2	4.83	0.96–24.1	0.05			
Albumin < 3.5 g/dL	2.8	0.62–10.7	0.12			
Hemoglobin < 12 g/dL	6.97	0.88–55.1	0.06			
LDH > ULN	1.06	0.19–5.84	0.93			
CCI > 2	5.0	1.00–24.9	0.04			
IPI > 4	3.50	0.06–19.2	0.14			
CD5 positive	1.29	0.15–11.1	0.81			
non GCB	0.10	0.01–0.96	0.04			
Simplified Frailty score frail	3.39	1.16–9.96	0.03	2.59	0.75–8.46	0.37
GNRI < 82	9.00	0.01–0.81	0.03	2.02	0.01–2.23	0.19
ARDI < 70	0.35	1.08–3.33	0.01	0.62	0.12–3.07	0.55

ARDI *average relative dose intensity*

Table 3 lists all the reported adverse events. The most common grade 3 or 4 adverse events were neutropenia (32%), febrile neutropenia (32%), and fatigue (21%). Serious adverse events occurred in 20 patients (53%). Adverse event-related deaths were reported in three patients (8%): one case of bacterial sepsis in a patient with cirrhosis, one case of COVID-19, and one case of sudden death of unknown origin involving a patient on dialysis. Notably, none of the patients experienced peripheral neuropathy (PN), and dose reduction due to adverse events was not required.

**Table 3:** Adverse events during treatment

Adverse events	n	%	Grade 3–5	%
neutropenia	21	(50)	12	(32)
anemia	17	(44)	5	(13)
thrombocytopenia	11	(28)	4	(10)
febrile neutropenia	12	(32)	12	(32)
bacterial pneumoniae	3	(8)	2	(5)
COVID-19	4	(11)	1	(3)
fatigue	15	(39)	8	(21)
peripheral neuropathy	0	(0)	0	(0)
liver dysfunction	0	(0)	0	(0)
renal dysfunction	1	(3)	1	(3)
sudden death of unknown origin	1	(3)	1	(3)
Septic shock	1	(3)	1	(3)
gastric ulcer	1	(3)	1	(3)
herpes zoster	1	(3)	1	(3)

None of the 16 patients who did not receive prophylactic G-CSF developed grade 3 or higher neutropenia or FN, likely due to reduced doses of doxorubicin and cyclophosphamide, which resulted in a mild degree of myelosuppression. However, there was no difference in ARDI between patients who received G-CSF and those who did not (received G-CSF vs. not received G-CSF; ARDI 66% vs. 62%, *p* = 0.297). In addition, several patients were hospitalized until they reached the myelosuppressive phase of the first course of pola-R-CHP, with some receiving peg-GCSF preparations from the first course, supported by healthcare services provided by health insurance agencies in Japan. All cases of FN occurred during the first course of treatment. Based on this retrospective analysis, among the 12 patients who developed FN, four experienced recurrent FN despite being treated with Peg-GCSF.

Table 4 lists grade 3 or worse treatment-related toxicities reported more frequently in the frail group than in the fit and unfit groups. Anemia and treatment mortality were higher in the frail group than in the other groups.

**Table 4:** Treatment-related toxicity by simplified frailty score

	**Simplified frailty score** **Frail (*n* = 19)**	**Fit or Unfit**(***n*** = 19)	*P* value
Hematologic toxicity, grade ≥ 3			
Anemia	5 (25.3%)	0	0.046
Neutropenia	10 (52.6%)	4 (21.1%)	0.091
Thrombocytopenia	4 (21.1%)	0	0.105
Febrile neutropenia, grade ≥ 3	8 (42.1%)	4 (21.1)	0.295
Any non-hematologic toxicity, grade ≥ 3	11 (57.9%)	7 (36.8%)	0.330
Treatment-related mortality	5 (26.3%)	0	0.046
Early treatment discontinuation	4 (21.1%)	0	0.105

## Discussion

In the POLARIX trial, the 1-year PFS rate was 83.9% [3]. In our retrospective study involving patients aged > 80 years with DLBCL treated with pola-R-CHP, the 12-month PFS rate was 78.5%. Although this study differs from the POLARIX trial in that all patients were aged > 80 years, presented with dose reduction, and included frailer patients who had a PS of ≥ 2 than the previous trials, the PFS observed in our study was not significantly lower than that observed in the POLARIX trial [3]. In addition, two previous phase II trials demonstrated the efficacy and safety of immunochemotherapy with mini-CHOP (approximately 50% RDI) in patients aged > 80 years, with reported 2-year survival rates of 59% and 64%, respectively [4, 5]. Our results suggest that age alone should not be a contraindication for pola-R-CHOP treatment in clinical practice and highlight the importance of an objective geriatric assessment for individualizing treatment intensity in this patient group. According to the prospective GERIAD study [11], approximately one-third of unfit older patients were unable to complete even three cycles of R-CHOP due to severe treatment-related toxicities, leading to early treatment discontinuation and, in some cases, early mortality. The results of this study are more favorable than those of some prospective reports, which may have been overestimated due to selection bias by excluding older patients with DLBCL, especially the frail patient population. This distinction is partly due to the retrospective study with a small number of patients.

Furthermore, various studies have explored the relationship between RDI and reduced chemotherapy with R-CHOP. For instance, Lee et al. conducted a retrospective multicenter analysis of 127 patients with DLBCL aged > 80 years treated with R-CHOP and found that the 2-year survival rate was significantly higher in the > 50% ARDI group (50.8% vs. 61.8%, *p* = 0.01) [12]. It was concluded that maintaining RDI as much as possible in R-CHOP therapy for very old patients aged > 80 years with DLBCL also decreased mortality risk. In our study, the 12-month OS was significantly higher in patients who maintained ARDI ≥ 70%. In contrast, new treatment strategies and intensive supportive care may be justified in patients with frailty. Therefore, patients with frailty who have more difficulty in maintaining ARDI may experience severe adverse events, even with low ARDI, and may have difficulty continuing treatment, thereby exhibiting a poorer effect on prolonging PFS.

However, the frequencies of neutropenia (32%) and febrile neutropenia (32%) in our study were higher than those reported in the POLARIX trial (30.8% and 14.3% [3], respectively). Patients in the frail group experienced a higher likelihood of serious adverse events even with reduced treatment intensity. This has also been reported for R-CHOP therapy in patients aged > 80 years [11, 12]. Physicians should consider ARDI appropriately depending on their clinical assessment of the risk of severe adverse event based on the frailty of the patient. In addition, to prevent infections, prophylactic measures such as peg-G-CSF administration, thorough early dose adjustments, and antimicrobials to prevent bacterial infections should be considered in very old patients. Owing to the reduced doses of doxorubicin and cyclophosphamide, prophylactic G-CSF administration is unnecessary in many cases. However, the study did not clarify whether, and to what extent, chemotherapy doses were reduced after G-CSF administration. In older patients, the risk of fatal FN is high; therefore, prophylactic G-CSF should be administered even when the chemotherapy dose is reduced. Fatigue was a more common adverse event in this trial than in the POLARIX study. In addition, the GERIAD trial showed that fatigue was the most common cause of difficulty in continuing R-CHOP therapy in older patients [11]. Fatigue is a common adverse event in the older population. Notably, none of the cases required a polar dose reduction owing to PN, unlike R-CHOP, where the vincristine dose had to be reduced owing to PN [13], impacting the dose intensity and potential quality of life. The frequency of PN was lower than that reported in the POLARIX trial. The risk of developing PN with Pola is associated with body weight (> 80 kg) and hypoalbuminemia (Alb < 3.5 g/dL). Although hypoalbuminemia was more common in this report than in the POLARIX study, the median albumin level in this study was 3.2 g/dL (2.1–4.5) vs 3.7 g/dL (1.7–5.4) in the POLARIX trial. However, weight was heavier in POLARIX: 51.0 kg (36–76) in this study vs. 74.2 kg (38.4–228.0) in the POLARIX trial [3, 14, 15]. Although this retrospective analysis has limitations, the study population comprised older Japanese individuals, who are smaller in body size than the Western population, and the large difference in body weight may have affected PN frequency. Further studies are warranted to compare the effects of pola-R-CHP and R-CHOP.

The limitations of this study include the small sample size, short follow-up period, and single-center study design. In addition, this was a retrospective study, which may have overestimated outcomes due to selection bias and may not accurately reflect the broader real population of older patients with DLBCL, especially patients with frailty. Further prospective studies involving multiple centers and extended follow-up periods are required to confirm the efficacy and safety of reduced pola-R-CHP therapy in older patients. In addition, this retrospective study with a small number of cases may have overestimated outcomes due to selection bias that does not accurately reflect the broad real-world population of older patients with DLBCL, especially those who are frail. In addition, the cost-effectiveness of substituting vincristine with Pola in older patients warrants further investigation. While factors such as medical costs and the need for careful and intensive management pose challenges, these approaches may enable older patients to receive sufficient RDI. Although the findings of this study may not be directly applicable to other countries, our study, conducted in Japan—the most advanced “super-aged” society worldwide—provides insights that can inform future treatment strategies for DLBCL in other developed nations. Ongoing clinical trials involving Pola in various aggressive lymphomas are expected to provide further benefits for older patients in the future [16].

In conclusion, pola-R-CHP may provide an effective treatment option for patients aged ≥ 80 years who are newly diagnosed with DLBCL. While dose reductions of chemotherapeutic agents may be necessary for safety reasons, maintaining the highest possible RDI to preserve treatment efficacy is crucial.

## Data Availability

No datasets were generated or analysed during the current study.
